# Astrocytes, emerging stars of energy homeostasis

**DOI:** 10.15698/cst2018.10.157

**Published:** 2018-09-29

**Authors:** Simonetta Camandola

**Affiliations:** 1 Laboratory of Neurosciences, National Institute on Aging, Intramural Research Program, Baltimore, MD, USA.

**Keywords:** astrocytes, hypothalamus, metabolism

## Abstract

Astrocytes have historically been considered structural supporting cells for neurons. Thanks to new molecular tools, allowing specific cell ablation or over-expression of genes, new unexpected astrocytic functions have recently been unveiled. This review focus on emerging groundbreaking findings showing that hypothalamic astrocytes are pivotal for the regulation of whole body energy homeostasis. Hypothalamic astrocytes sense glucose and fatty acids, and express receptors for several peripheral hormones such as leptin and insulin. Furthermore, they display striking sexual dimorphism which may account, at least partially, for gender specific differences in energy homeostasis. Metabolic alterations have been shown to influence the initiation and progression of many neurodegenerative disorders. A better understanding of the roles and interplay between the different brain cells in regulating energy homeostasis could help develop new therapeutic strategies to prevent or cure neurodegenerative disorders.

## INTRODUCTION

The brain is the organ responsible for the centralized control of the other organs’ functions and, in higher vertebrates, of reasoning. Such tasks are achieved thanks to the interconnections of billions of neurons and glia cells. Due to their role in receiving, processing, and transmitting information neurons are considered to be the primary cell types of the central nervous system, and the only repository of reasoning and awareness. Astrocytes are the most abundant type of glial cells 1. Given their ‘not electrically excitable’ nature, it has long been assumed that astrocytes merely act as structural supporting cells for neurons. However, phylogenetic and recent experimental evidence corroborate the idea that glia in general, and astrocytes in particular, may have played a direct role in the establishment of higher cognitive function. While the rat cerebral cortex contains roughly 0.4 glia to neuron cells 2, the human highly evolved neocortex has a ratio of 1.4 3,4. The same overall increase is also observed within a species based on the evolutionary stage 2,5, and the neuronal density of the brain area considered 6. Rather than to brain size the change seems to correlate to the average neuronal size and is independent from neuronal metabolic requirements 6. While the number of neurons is not proportional to the size of the brain, there is an almost linear correlation amongst non-neuronal cell density and brain mass in species spanning 90 million of years of evolution 7. This suggests the existence of an evolutionary conserved mechanism to add glial cells to the brain as its size and/or complexity increased 8. It also infers that the functions of glial cells are so fundamental for brain physiology to have been maintained for millions of years of evolution 6. It was recently shown that the engraftment of mice with human derived astrocytes increases activity dependent plasticity and learning compared to the rodent engrafted counterparts 9. Alterations in astrocyte function result in impairment of slow wave synchronicity and sleep 10, memory consolidation 11, and associative memory 12, strongly substantiating their involvement in rhythm generation and neural network patterning. At a cellular level, it has been established that astrocytes are instrumental for modulating synaptic plasticity by regulating the formation, maintenance and removal of synapses 13, the rate of local cerebral blood flow and the volume of the extracellular space 14, the secretion or removal of neurotransmitters from the synaptic cleft 15,16. Furthermore, brain energy production, delivery and storage heavily rely on the astrocytic repertoire of receptors, channels, transporters, and enzymes. The wealth of progress in the understanding of the bidirectional metabolic coupling between neurons and astrocytes, and its impact on brain metabolism and cognition has been extensively reviewed over the past years 17,18,19,20,21. This short review will focus on emerging evidence showing that astrocytes are not only essential for brain function and metabolism, but also contribute to whole body energy homeostasis.

## HYPOTHALAMIC ASTROCYTES AS NUTRIENT SENSORS

The hypothalamus is the portion of the brain that integrates sensory inputs from the external environment with hormonal and neural signals from the body, allowing ad hoc short- and long-term homeostatic adjustments 22. As a result of the coordinated output of several neuronal networks, the hypothalamus is involved in the control of satiety and hunger, body temperature, sleep, circadian rhythms, thirst 23,24,25. From an anatomical point of view, the hypothalamus shows unique features compared to the rest of the brain. The medial-ventral portion of the hypothalamus (i.e. median eminence, vascular organ of the lamina terminals, subfornical organ) is characterized by the absence of blood-brain barrier and extensive vascularization with highly fenestrate capillaries 26,27. Similar, fenestrated capillarization is also observed in the ventromedial area and the nucleus arcuate 28,29. This peculiar vascularization bestows these areas the ability to release hypothalamic hormones in the general blood stream, as well as the chance to act as sensory hubs. An intricate network of specialized glial cells, tanycytes and astrocytes, regulates the diffusion and response to circulating factors such as peripheral hormones, nutrients and peptides. Glucose is the main energy source of the mammalian brain and astrocytes actively coordinate its uptake, metabolization and storage 19,20,21. In the hypothalamus the role of the neuron-astrocyte functional glucose coupling expands beyond the fulfillment of energy requirements. Hypothalamic astrocytes actively cooperate with specialized ‘glucose sensitive' neurons 30 in detecting circulating glucose levels, and generating the proper systemic metabolic response. This is suggested by experimental evidence showing that the expression of glucose transporters GLUT1 and GLUT2 in astrocytes is critical for glucose sensing. In rodents, whole body as well as hypothalamic hyperglycemia impairs glucose sensing by lowering the expression of GLUT1 in astrocytes 31. However, the systemic glucose lowering response induced by hyperglycemia is restored by specific adenovirus-mediated re-expression of GLUT1 in hypothalamic astrocytes 31. Animals expressing a GLUT2 dominant negative construct that prevents glucose sensing but retains intact transport capacities, are hyperphagic with altered hypothalamic orexin, thyrotropin-releasing hormone and corticotropin-releasing hormone expression 32. Similarly, GLUT2 knock out mice rescued by pancreatic expression of GLUT1 display temporal anomalies in fasting-refeeding behaviors, impaired hypothalamic orexigenic and anorexigenic neuropeptide expression, and impaired systemic response to a glucose challenge 33. In genetic complementation experiments, the specific re-expression of GLUT2 in astrocytes, but not in neurons, was able to restore the normal glucagon secretion response to physiological hypoglycemia 34. Further evidence of the requirement of astrocytes in proper glucose sensing comes from studies using knock out models for connexins. Astrocytes are tightly connected by gap junctions to form a large functional syncytium that allows the selective transmission of nutrients and signaling molecules over long distances 35. Connexins form hemi-channels that are instrumental for astrocytic network transmission. The siRNA-mediated ablation of connexin 43 in the arcuate nucleus diminishes the release of insulin from the pancreas in response to central glucose upregulation 36. This finding supports the notion that astrocytic intercommunication is essential to proper central glucose sensing and peripheral response 36. Under conditions of reduced glucose availability, the body can fulfill its energy requirements by switching from glucose to fatty acids utilization. In the brain, astrocytes are the only cell type able to utilize fatty acids for the synthesis of ketones bodies 37. Contrary to most brain regions the production of ketones in the hypothalamus is relatively high and, at least for certain fatty acids, glucose dependent 38,39. The oxidation of palmitate, but not oleate, is indeed decreased by glucose via 5’-adenosine monophosphate-activated protein kinase (AMPK)-dependent mechanism in hypothalamic but not cortical brain slices 39. Hypothalamic ketones levels are important to mediate food behavior. Specifically, high ketones production in the arcuate nucleus and ventromedial hypothalamus, signals high fat intake and elicits a reduction in caloric intake 40. Furthermore, the direct actions of glucose and free fatty acids on sensing neurons in the ventromedial hypothalamus can be overrode by ketones released from astrocytes 40. Another example of involvement of astrocytes in lipid sensing is given by the observation that intraventricular infusion of apolipoprotein E (ApoE) decreases food intake, while its neutralization with anti-ApoE antibodies stimulates it 41. Astrocytes are the brain predominant site of cholesterol synthesis 42 and the principal cell type expressing ApoE, thus the most likely effectors of ApoE-mediated food behaviors 41,43. Interestingly, both ketones and ApoE have been linked to central leptin signaling. Acetoacetyl-CoA synthase, a neuronal ketone body utilizing enzyme, is selectively induced by leptin in the ventromedial hypothalamus and arcuate nucleus via AMPK inhibition 44. Leptin can also upregulate ApoE levels 43.

## ASTROCYTES AS MEDIATORS OF ENDOCRINE SIGNALING 

The possibility that astrocytes may participate in the regulation of body homeostasis in other ways than direct nutrient sensing is inferred by the fact that they express receptors for hormones involved in energy homeostasis such as leptin 45,46, ghrelin 47, insulin-like growth factor-1 48, thyroid hormone 49, glucagon like peptide-1 GLP-1 50, and insulin 51. How the specific activation of these receptors in hypothalamic astrocytes impacts metabolism has just began to be explored. Morphological and biochemical changes have been shown in hypothalamic astrocytes following hormonal stimulation. For example, changes in circulating levels of leptin modify the expression of astrocytic glucose and glutamate transporters 52,53, as well as the extension of sheathing and synaptic contacts on adjacent neurons 52,54,55. These structural changes result in altered neuronal electrophysiological responses. The astrocytic-specific ablation of the leptin receptor leads to decreased astrocytic projections and coverage of proopiomelanocortin neurons, modulation of the electrical activity of proopiomelanocortin and agouti-regulated protein neurons, and ultimately in the attenuation of the anorexigenic response 55,56. While under normal feeding conditions astrocytic-deficient leptin receptor mice do not show a clear metabolic phenotype 55,57 when challenged with a high fat diet they are partially protected from hyperleptinemia and leptin resistance thus obesity 57. Consistently, diet-induced obese animals show an increase of hypothalamic astrocytic coverage, as well as specific increase of leptin receptor in glial fibrillary acid protein (GFAP)- positive cells 58. It has also been suggested that leptin-mediated satiety effects could be facilitated by astrocytes responses to ghrelin. Stimulation of astrocytes with ghrelin modify glutamate and glucose metabolism as well as glycogen storage by decreasing GLUT2, glutamine synthetase and lactate dehydrogenase, and increasing glutamate uptake, glycogen phosphorylase and lactate transporters 47. Furthermore, ghrelin-mediated increase in food intake could be suppressed by the release of adenosine from activated astrocytes and consequent adenosine receptor A1-mediated inhibition of agouti-related peptide producing neurons in the nucleus arcuate 59,60. Despite the presence of insulin sensitive glucose transporters GLUT4 and GLUT8 in various areas 21, contrary to peripheral organs, brain glucose fluxes are considered insulin-independent 61,62. However, there is evidence that insulin signaling modulates central and systemic metabolic homeostasis 63,64. The mechanisms of central insulin actions are still poorly understood. It was recently shown that hypothalamic astrocytes insulin signaling is essential to integrate central glucose sensing and systemic glucose metabolism 50. Utilizing several glial-specific loss of function models Garcia-Caceres and colleagues 50, elegantly demonstrated that the postnatal ablation of insulin receptor in hypothalamic astrocytes modify their morphology, mitochondrial function, and connectivity, resulting in reduced glucose dependent-activation of proopiomelanocortin neurons and impaired systemic response to changes in glycemia.

## HYPOTHALAMIC ASTROCYTES SEXUAL DIMORPHISM

Neurological and neurodegenerative disorders are often characterized by sexual dimorphism in terms of either incidence or severity and progression of the pathology 65. Sexual dimorphisms at both morphological and physiological levels have been reported for several areas of the brain including cortex, hippocampus, amygdala and hypothalamus 66,67,68. In the hypothalamus the morphological changes can be appreciated even in gross anatomy and are particularly striking in astrocytes. Female’s astrocytes have a simple bipolar structure rather than the complex stellate shape found in males 69. Furthermore, the levels of GFAP immunostaining in the arcuate nucleus of males increases from birth throughout adulthood 70. The presence of receptors for estrogen, androgen and progesterone 71,72,73 is believed to underlie the dynamic morphological changes and possible different functional responses of hypothalamic astrocytes in males and females 72,74. In the preoptic area the levels of astrocytic connexin 43 are regulated by estrogen and progesterone in a sex specific manner 75. Glial structural changes are seen in the hypothalamic areas both in rodents and humans during the estrous cycle 76,77. In non-human primates the glial coverage on gonadotropin-releasing hormone (GnRH) secreting neurons increases, while the number of synaptic contacts decreases following ovariectomy, a phenomenon that can be reverted by estrogen replacement 78. Mechanistically the steroid-induced morphology changes are due to increased (-aminobutyric acid (GABA) signaling via GABA_A_ receptors 79. The impact of sex hormones on metabolism regulation is well known. Estrogen receptor alpha (ERa) regulates food intake, glucose homeostasis and augments energy expenditure 80,81. Fluctuation in food intake is seen in females based on their menstrual cycle, with the lowest during the preovulatory phase when estrogen peaks 82,83. Conversely, higher energy intake and increased fat consumption are seen during the progesterone-controlled luteal phase 82,83. The emerging roles of astrocytes in body energy homeostasis and their clear sexual dimorphism, suggest that they may be instrumental in regulating the different responses of females and males to dietary challenges 84,85. Supporting such hypothesis is the evidence that hypothalamic astrogliosis and inflammation following high fat diet are higher in male rodents compared to females 85,86. *In vitro* experiments show different responses to saturated fatty acids in astrocytes isolated from males and females 86,87. *In vivo*, long term high fat diet increases the levels of estradiol in females, while in males its levels are unchanged and associated with a significant decrease of ERa in the hypothalamus 87. As ERa activation by estrogen have been shown to protect females from diet induced-obesity 80,88, it is possible that astrocytic hypothalamic ERa levels are pivotal in mediating such differences 87. Notably, the discrepant hypothalamic inflammatory responses observed in wild type animals following an obesogenic diet 86 are lost in ERa knockout animals 86,88. Consistent with their ability to synthetize cholesterol 42, astrocytes are also the primary steroidogenic cells in the brain 89. The most prevalent hypothalamic neurosteroids are progesterone and its derivative allopregnanolone 89,90. Interestingly, the synthesis of neuroprogesterone has been shown to be upregulated by estradiol in hypothalamic astrocytic cultures from females but not male rats 91. Experimental evidence suggests that in addition to the better characterized roles in sexual behavior, anxiety, analgesia and sleep 92,93,94,95,96, neurosteroids may influence energy homeostasis. Levels of allopregnanolone have been associated with changes in food intake and eating disorders in humans 82,96. In rats, administration of allopregnanolone increased the feeding latency, as well as the meal duration and the preference for fat 97,98. Although the specific mechanisms underlying the changes in feeding behavior have not been elucidated, neurosteroid’s best characterized molecular function is the modulation of GABA signaling at GABA_A_ receptors 99. Hypothalamic GABA transmission is instrumental for proper feeding behavior and energy homeostasis 100. As per other aspects of energy metabolism, recent evidence shows that astrocytes play a pivotal role in GABA-mediated food related behavior. Hypothalamic astrocytes morphology drastically changes in response to nutritional status 101. High-order astrocytic processes shorten during fasting and elongate during fed status 101. These dynamic changes are associated with modified GABA transmission in adjacent neurons and metabolic dysregulation 101.

## CONCLUSIONS

Metabolic alterations influence the initiation and progression of many neurodegenerative disorders 21. Clinical evidence shows pre- and early-symptomatic changes in the hypothalamus in patients with Alzheimer's disease, Parkinson's disease, Huntington's disease, and amyotrophic lateral sclerosis 102. Although the hypothalamus senses and regulates energy homeostasis, not many studies have explored the reciprocal influence of the different cell types in managing energy homeostasis, nor their involvement in the progression of neurodegenerative disorders. The neuron-centric and "most impacted area of the brain" focus, together with the over reliance on male animals in preclinical studies, have hindered the elucidation of the biological mechanisms that underly brain energy sensing and management in both genders. Understanding these underpinning biological differences by expanding our scientific focus, could be key in developing strategies for diagnosis and interventions.

## Abbreviations

ApoE: apolipoprotein E
ERα: estrogen receptor alpha
GABA: γ-aminobutyric acid
